# Critical role of metabotropic glutamate receptor 4 in bone marrow-derived dendritic cells in the Th17 cell differentiation and the melanogenesis of B16 cells

**DOI:** 10.1590/1414-431X20209282

**Published:** 2020-04-06

**Authors:** Guangming Zhao, Wenhui Zhou, Ying Liu, Yupeng Wang, Zhou Li, Zhiqi Song

**Affiliations:** 1 Department of Dermatology, The First Affiliated Hospital of Dalian Medical University, Dalian, Liaoning, China; 2 Department of Dermatology, The Second Xiangya Hospital of Central South University, Changsha, Hunan, China

**Keywords:** Metabotropic glutamate receptor-4, Th17, Melanogenesis, Vitiligo

## Abstract

Vitiligo is an acquired pigmentary disorder resulting from selective destruction of melanocytes. Emerging studies have suggested that T helper cell 17 (Th17) is potentially implicated in vitiligo development and progression. It was recently discovered that metabotropic glutamate receptor 4 (mGluR4) can modulate Th17-mediated adaptive immunity. However, the influence of mGluR4 on melanogenesis of melanocytes has yet to be elucidated. In the present study, we primarily cultured mouse bone marrow-derived dendritic cells (BMDC) and then knocked down and over-expressed mGluR4 using transfection. Transduced BMDC were co-cultured with CD4^+^ T cells and the expression of Th17-related cytokines were measured. The morphology and melanogenesis of B16 cells were observed after being treated with co-culture medium of CD4^+^ T cells and transduced BMDC. We found that mGluR4 knockdown did not affect the co-stimulatory CD80 and CD86 upregulation after lipopolysaccharide stimulation but did increase the expression of Th17-related cytokines, and further down-regulated the expression of microphthalmia-associated transcription factor (MITF) and the downstream genes, decreased melanin production, and destroyed the morphology of B16 cells. Conversely, over-expression of mGluR4 reduced the expression of CD80 and CD86, suppressed the production of Th17-related cytokines, increased the expression of MITF, and did not destroy the morphology of B16 cells. Our study confirmed that mGluR4 modulated the Th17 cell polarization and resulted in the alteration of melanogenesis and morphology of B16 cells. Collectively, these findings suggest mGluR4 might be a potent target involved in the immune pathogenesis of vitiligo.

## Introduction

Affecting 0.5% of the world's population, vitiligo is an acquired pigmentary disorder resulting from selective destruction of melanocytes. Genetic predisposition, autoimmune mechanism, and oxidative stress are generally recognized as the major pathophysiological causes for the loss of functional melanocytes (1). Currently, a growing amount of evidence supports the hypothesis of autoimmune etiology. Emerging studies suggest that T helper cell 17 (Th17) is potentially involved in vitiligo development and progression (2–5). It has been proven that serum IL-17 levels and tissue IL-17A mRNA levels of skin biopsies are elevated in vitiligo patients (2–5). There are also significant positive correlations between IL-17 level and the activity, extent, and severity of vitiligo (6). Sushama et al. (7) reported that cytokines secreted by Th17 cells played a crucial role in the maintenance and spread of vitiligo, since they were increased in accordance with the extent of disease.

Dendritic cells (DC) are antigen presenting cells (APC) that bridge the crosstalk between innate and adaptive immunity (8). They provide the antigenic, costimulatory, and cytokine signals to drive T cell differentiation (9). Costimulatory molecules CD40, CD80, and CD86 are expressed on DC, leading to the secretion of cytokines and stimulation of the immune response. The differentiation of naive CD4^+^ T helper (Th) cells into effector cells mainly depends on cytokines produced by DC. In response to TGF-β together with IL-6 and IL-23, naive T cells differentiate into Th17 cells, which mediate immunity against fungi and extracellular bacteria (10). Th17 cells produce the signature cytokines IL-17A, IL-17F, and IL-22. Retinoic acid-related organ receptors (ROR) γt and RORα are the lineage-specific transcription factors essential for Th17 cell differentiation, while RORγt plays a more important role in specifying the Th17 cell fate (11).

Metabotropic glutamate receptor 4 (mGluR4), a member of group III mGluRs, is mainly a presynaptic receptor and belongs to family C G-protein-coupled receptors. mGluR4 is expressed in cerebellar granule cell neuro-precursors and embryonic stem cell-derived neural progenitor/stem cells, and is probably effective in suppressing cell proliferation and inducing neuronal differentiation (12–14). Recently, Fallarino et al. (15) reported that mGluR4-knockout mice were dramatically susceptible to experimental autoimmune encephalomyelitis (EAE, a mouse model of multiple sclerosis) and initiated immune responses dominated by Th17 cells. Wild-type mice treated with the mGluR4-positive allosteric modulator N-phenyl-7-(hydroxyimino) cyclopropa[b]chromen-1a-carboxamide (PHCCC) significantly attenuated the disease due to increased expression of regulatory T cells and reduced levels of Th17 cells. These findings showed that mGluR4 could modulate Th17-mediated adaptive immunity. However, there is little evidence regarding the role of mGluR4 pathway in the Th17-mediated immune pathogenesis of vitiligo.

In the present study, we explored whether mGluR4 participated in the modulation of melanogenesis by affecting Th17 differentiation. Our research provided new evidence for how mGluR4 signaling influenced the Th17-mediated immune pathogenesis of vitiligo at the molecular level, which will facilitate developing novel and targeted therapies for vitiligo.

## Material and Methods

### Mice

Male C57BL/6 mice and BALB/c mice, all aged 4–8 weeks, were purchased from Center of Experimental Animals, Dalian Medical University, China. Mice were bred five per cage in an isolated specific pathogen-free environment. All the animal experiments were carried out in strict accordance with the institutional guidelines for the ethical treatment of laboratory animals. The protocol was approved by the Committee on the Ethics of Laboratory Animals of Dalian Medical University (Permit Number: L2015012).

### Isolation of mouse bone marrow-derived dendritic cells (BMDC)

Bone marrow was flushed from the femurs and tibiae of C57BL/6 mice and depleted of red blood cells (RBC) with RBC Lysing buffer (Solarbio, China). Cells were seeded in 6-well plates (1×10^6^ cells/mL; 2 mL/well) in RPMI 1640 medium supplemented with 10% FBS (Gibco/BRL, Thermo Fisher Scientific, USA), 100 U/mL penicillin, 100 mg/mL streptomycin, 10 ng/mL granulocyte-macrophage colony-stimulating factor (GM-CSF, PeproTech Inc., USA), and 10 ng/mL IL-4 (PeproTech Inc.) at 37°C in a humidified 5% CO_2_ atmosphere. Half of the old medium was discarded every other day and substituted for fresh BMDC medium with cytokines. Lipopolysaccharide (LPS) (1 µg/mL, Sigma, USA) was added on day 6 to stimulate maturation and cells were cultured for an additional 24 h to obtain mature BMDC.

### siRNA transfection

For transient transfection experiments, three predesigned mGluR4-targeted siRNAs and negative control siRNA (NC) (GenePharma Co., China) were diluted with Opti-MEM (Gibco/BRL, Thermo fisher Scientific, USA) and transfected into cells using INTERFERin transfection reagent (Polyplus Transfection, France) according to the manufacturer's instructions. Transfected cells were incubated at 37°C in a 5% CO_2_ incubator for 24 h.

### Flow cytometric analysis

DC of each group were collected and incubated with PE-labeled-anti-CD80 antibody, APC-labeled-anti-CD86 antibody (BD Biosciences, USA) or isotype-matched control antibodies for 30 min at 4°C. After incubation, cells were washed twice with PBS, and then fixed with 2% paraformaldehyde. Cellular fluorescence intensity was determined by FACScan (BD Biosciences). Data were analyzed by FlowJo software (Treestar Inc., USA), by calculating the percentage of positive cells compared to the isotype controls.

### Allogeneic mixed lymphocyte reaction

Splenocytes were isolated from male BALB/c mice. CD4^+^ T cells were purified from splenocytes via positive selection using mouse CD4 (L3T4) MicroBeads and a MiniMACS™ Starting kit (Miltenyi Biotec, Germany). Purity of CD4^+^ T cells was assessed to be >92% by flow cytometry after stained with FITC-conjugated anti-CD4 Ab. DC pretreated by mitomycin C (25 μg/mL; Roche Inc., Switzerland) for 30 min were washed with PBS, and then were co-cultured with 2×10^5^ purified allogeneic T cells. The ratio of DC:T cells was 1:1, because this ratio exerted the most significant effect of stimulating allogeneic T cell proliferation as proven by our previous research (16).

### RNA isolation and qRT-PCR

Total mRNA was extracted with RNAiso-Plus (Takara Bio Inc., Japan). cDNA synthesis was performed with PrimeScript™ RT reagent kit with gDNA Eraser (Takara Bio Inc.). qRT-PCR was performed on the StepOnePlus^TM^ Real-Time PCR System (Life Technologies, USA). The primers containing mGluR4 siRNA-1 (forward 5′-CCUACUCCUCAGCCUUUAUTT-3′ and reverse 5′-AUAAAGGCUGAGGAGUAGGTT-3′); mGluR4 siRNA-2 (forward 5′-GCUCCCGACUUGAGUGAUATT-3′ and reverse 5′-UAUCACUCAAGUCGGGAGCTT-3′); mGluR4 siRNA-3 (forward 5′-GCCUUCUGGAAACGUCCAATT-3′ and reverse 5′-UUGGACGUUUCCAGAAGGCTT-3′); IL-6 (forward 5′-CCACTTCACAAGTCGGAGGCTTA-3′ and reverse 5′-CCAGTTTGGTAGCATCCATCATTTC-3′); IL-23a (forward 5′-GGAAAGCTGGACCACCACA-3′ and reverse 5′-CTTTGAAGATGTCAGAGTCAAGCAG-3′); IL-17A (forward 5′-GGCCCTCAAGTGGAACTATG-3′ and reverse 5′-CACACCCACCAGCATCTTCTC-3′); RORγt (forward 5′-TCTGCAAGACTCATCGACAAGG-3′ and reverse 5′-CACATGTTGGCTGCACAGG-3′); MITF (forward 5′-CAGCCCTATGGCTATGCTCACTC-3′ and reverse 5′-GTGTTCATACCTGGGCACTCACTC-3′); tyrosinase (TYR) (forward 5′-CAAGTACAGGGATCGGCCAAC-3′ and reverse 5′-GGTGCATTGGCTTCTGGGTAA-3′); tyrosinase-related protein 1 (TRP-1) (forward 5′-TGATGCGGTCTTTGACGAATG-3′ and reverse 5′-GTTGGTAACTGGAGGCCAGAATG-3′); and GAPDH (forward 5′-GCCTTCCGTGTTCCTACCC-3′ and reverse 5′-CAGTGGGCCCTCAGATGC-3′) were purchased from Takara Bio Inc. Reactions were performed using SYBR^®^ Premix Ex Taq^TM^ II (Tli RNaseH Plus) (Takara Bio Inc.) designed primers and cDNA of template, or non-template control. Results were normalized to the housekeeping gene GAPDH.

### cDNA transfection

pcDNA3.1 mGluR4 cDNA and pcDNA3.1 control were synthesized by GenePharma Co. (China). They were transfected into BMDC using Effectene Transfection reagent (QIAGEN GmbH, Germany) according to manufacturer instructions. Transfected cells were incubated at 37°C in a 5% CO_2_ incubator for 24 h. Total RNA and protein were harvested separately and stored at −80°C until use.

### Western blotting

DC were lysed in RIPA lysis buffer (KeyGen Biotech, China) on ice for 30 min. A total of 40 µg proteins were subjected to 8% sodium dodecyl sulfate polyacrylamide gel electrophoresis and transferred onto polyvinylidene difluoride (PVDF) membranes (Millipore Co., USA). Membranes were blocked with 5% skimmed milk and incubated with antibodies to mGluR4 and β-actin overnight at 4°C. Then membranes were washed with TBST followed by incubation with secondary antibodies conjugated with horseradish peroxidase. The immune bands were visualized by the Western Bright™ ECL (Advansta Inc., USA) and subsequently analyzed for densitometry with AlphaView software for FluorChem™ Systems (ProteinSimple, USA). Results are reported as relative intensity and the relative expression of β-actin was used as a control.

### Culture, cell treatment, and microscopy of B16 cells

B16 cells were cultured in DMEM supplemented with 15% FBS, 100 U/mL penicillin, and 100 μg/mL streptomycin. After two passages, B16 cells were seeded at 2×10^5^ cells/well into 6-well plates for 24 h, and the medium was replaced with the co-culture medium of the transduced DC and CD4^+^ T cells. Melanocyte morphology was observed under a microscope (DMIL, Leica, Germany) 24 h later, and then cells were collected for mRNA extraction.

### Melanin content assay

Following incubation with the co-culture medium of the transduced DC and CD4^+^ T cells for 48 h, B16 cells were washed twice with PBS and the pellets of treated cells were dissolved in 1 M NaOH at 95°C for 1 h, and the concentration of melanin was calculated by measuring the absorbance at 405 nm (Biotek Instruments, USA).

### Statistical analysis

Data are reported as means±SD from three independent experiments. Statistical analyses were carried out by the SPSS software package (version 19.0; SPSS Inc., USA). One-way analysis of variance (ANOVA) was used for three or more groups. A P value <0.05 was considered to be statistically significant.

## Results

### siRNA transfection reduced mGluR4 expression levels in BMDC

**Figure 1:**
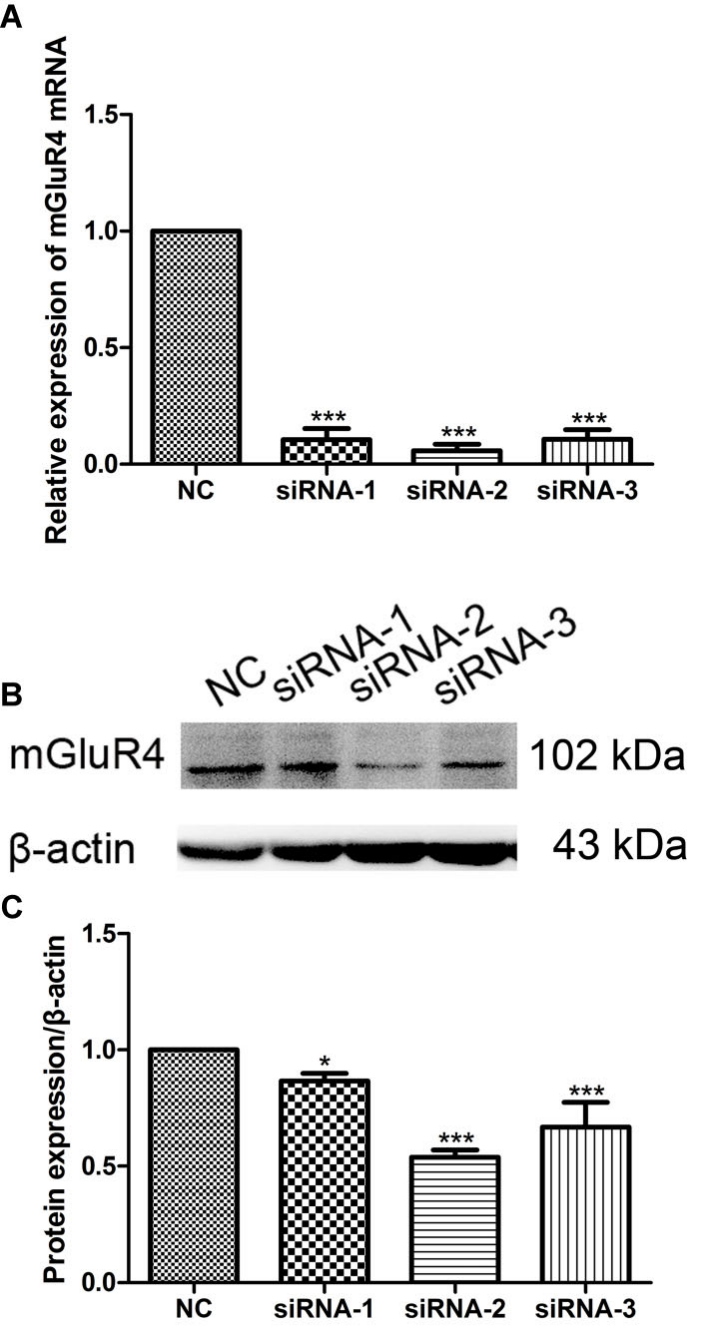
Interference efficiency of mGluR4 siRNA in bone marrow-derived dendritic cells (BMDC). Three mGluR4-targeted siRNAs were predesigned. Mouse BMDC were transfected with negative control siRNA (NC) or one of the three mGluR4 siRNAs (siRNA). Twenty-four hours later, the efficiency of silencing was detected by qRT-PCR (**A**) and western blotting analysis (**B**). **C**, Quantitative mGluR4/β-actin expression levels. Data are reported as means±SD. *P<0.05, ***P<0.001 *vs* NC (ANOVA).

mGluR4 siRNA transfection in BMDC down-regulated the expression of mGluR4 at both the mRNA and protein levels (Figure 1, P<0.001, P<0.05). siRNA-2 played the best role in decreasing the mGluR4 expression among the three designed siRNAs, and was therefore used for siRNA transfection in the following experiments. These results showed that we successfully knocked down the expression of mGluR4 in BMDC by siRNA transfection.

**Figure 2:**
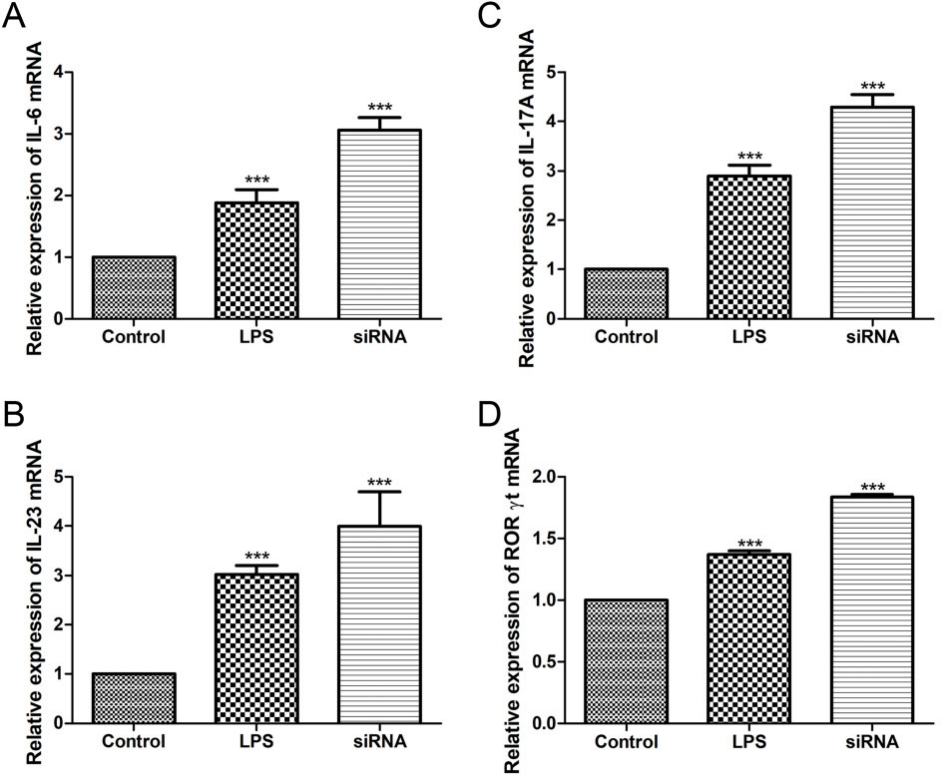
Th17 cell differentiation induced by mGluR4 knockdown in bone marrow-derived dendritic cells (BMDC). The relative mRNA expressions of interleukin (IL)-6 (**A**) and IL-23 (**B**) were evaluated by qRT-PCR in immature (Control), mature (lipopolysaccharide, LPS), and mGluR4 siRNA transfected (siRNA) BMDC. CD4^+^ T cells were co-cultured with the BMDC for 24 h at the ratio of 1:1. The relative mRNA expressions of IL-17A (**C**) and RORγt (**D**) in the co-culture environment were determined by qRT-PCR. Data are reported as means±SD. ***P<0.001 *vs* Control (ANOVA).

**Figure 3:**
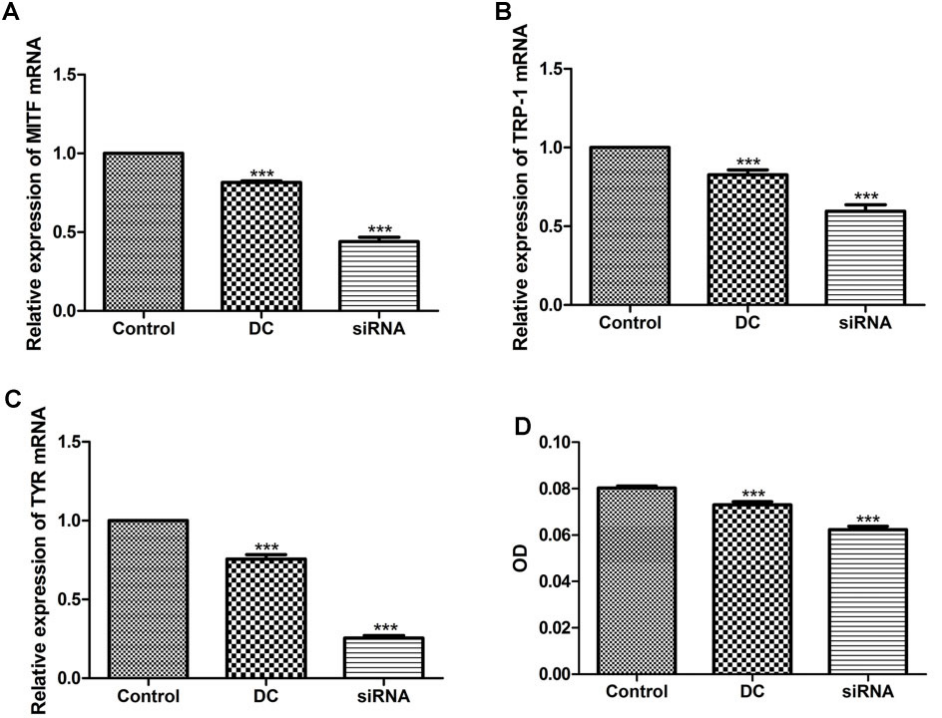
Effect of increased production of Th17-related cytokines on melanocyte function. B16 cells were treated with co-culture medium of CD4^+^ T cells and the negative control siRNA transduced dendritic cells (DC), mGluR4 siRNA transduced DC (siRNA), or only RIPM-1640 medium (Control). Twenty-four hours later, the mRNA expressions of MITF (**A**), TYR (**B**), and TRP-1 (**C**) were measured by qRT-PCR. **D**, Forty-eight hours later, B16 cells exposed to the different interventions were treated with 1 M NaOH and processed for absorbance (OD) at 405 nm. Data are reported as means±SD. ***P<0.001 *vs* Control (ANOVA).

**Figure 4:**
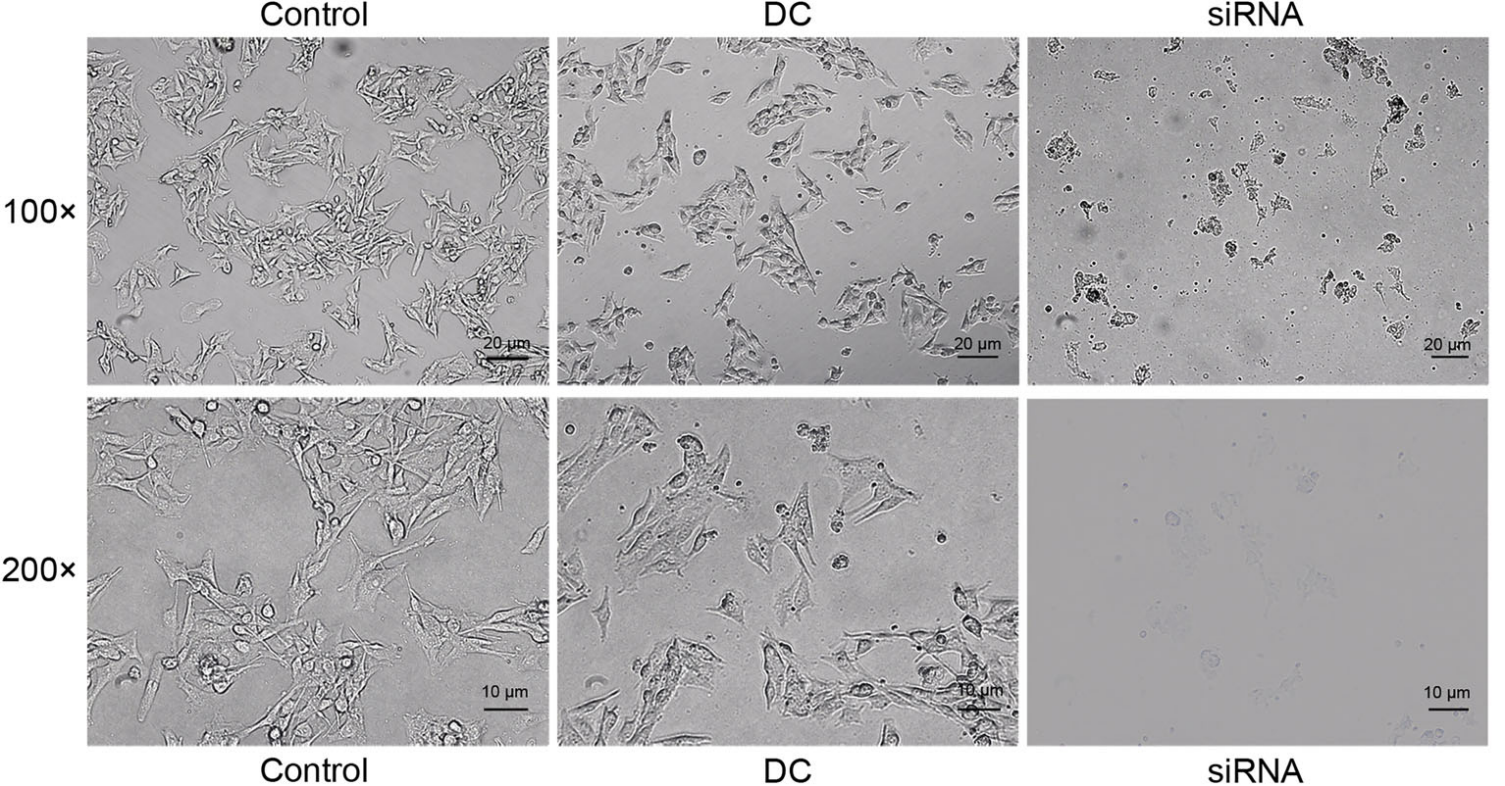
Effect of increased production of Th17-related cytokines on morphology of B16 cells. B16 cells were exposed to the co-culture medium of CD4^+^ T cells and the negative control siRNA transduced dendritic cells (DC), mGluR4 siRNA transduced DC (siRNA), or only RIPM-1640 medium (Control). Twenty-four hours later, melanocyte morphology was observed under a microscope with 100 and 200× magnification (scale bars: upper panels 20 µm; lower panels 10 µm). Representative images are shown.

**Figure 5:**
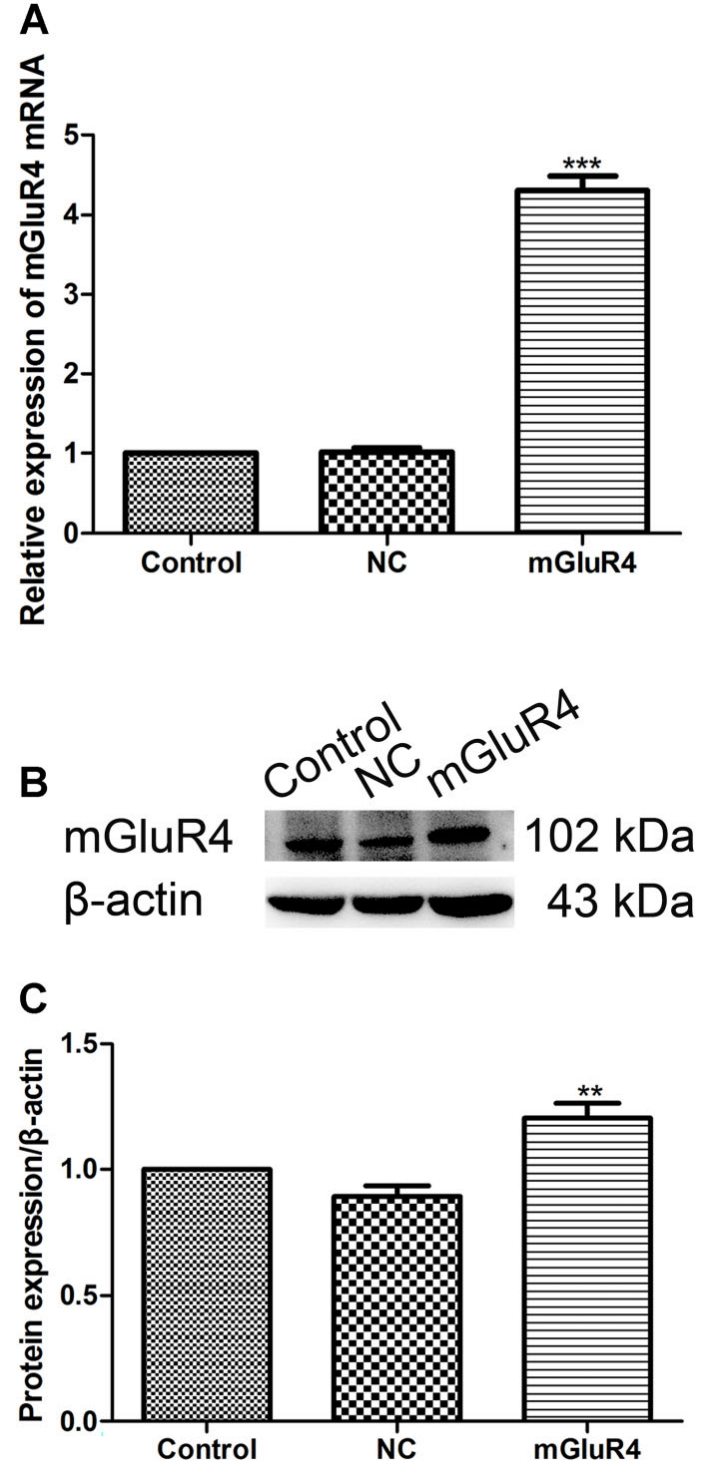
mGluR4 over-expression in bone marrow-derived dentritic cells (BMDC). BMDC were transfected with empty vector (negative control, NC), plasmid expressing mGluR4 (mGluR4), or untreated (Control). Twenty-four hours later, the expression of mGluR4 was determined by qRT-PCR (**A**) and western blotting analysis (**B**). **C**, Quantitative mGluR4/β-actin expression levels. Data are reported as means±SD. **P<0.01, ***P<0.001 *vs* Control (ANOVA).

**Figure 6:**
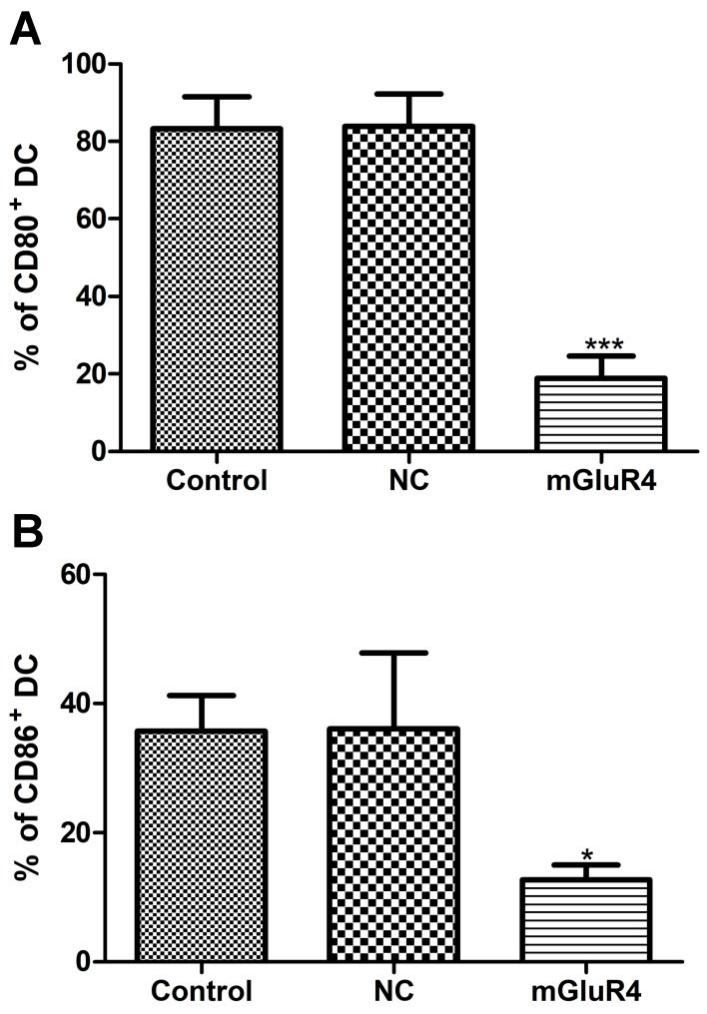
Effect of mGluR4 over-expression on surface marker expression of costimulatory molecules CD80 and CD86 in dendritic cells (DC). DC were transfected with empty vector (negative control, NC), plasmid expressing mGluR4 (mGluR4), or untreated (Control). Twenty-four hours later, cells were collected and the percent of DC expressing CD80 (**A**) and CD86 (**B**) was quantified by flow cytometry. Data are reported as means±SD. *P<0.05, ***P< 0.001 *vs* Control (ANOVA).

**Figure 7:**
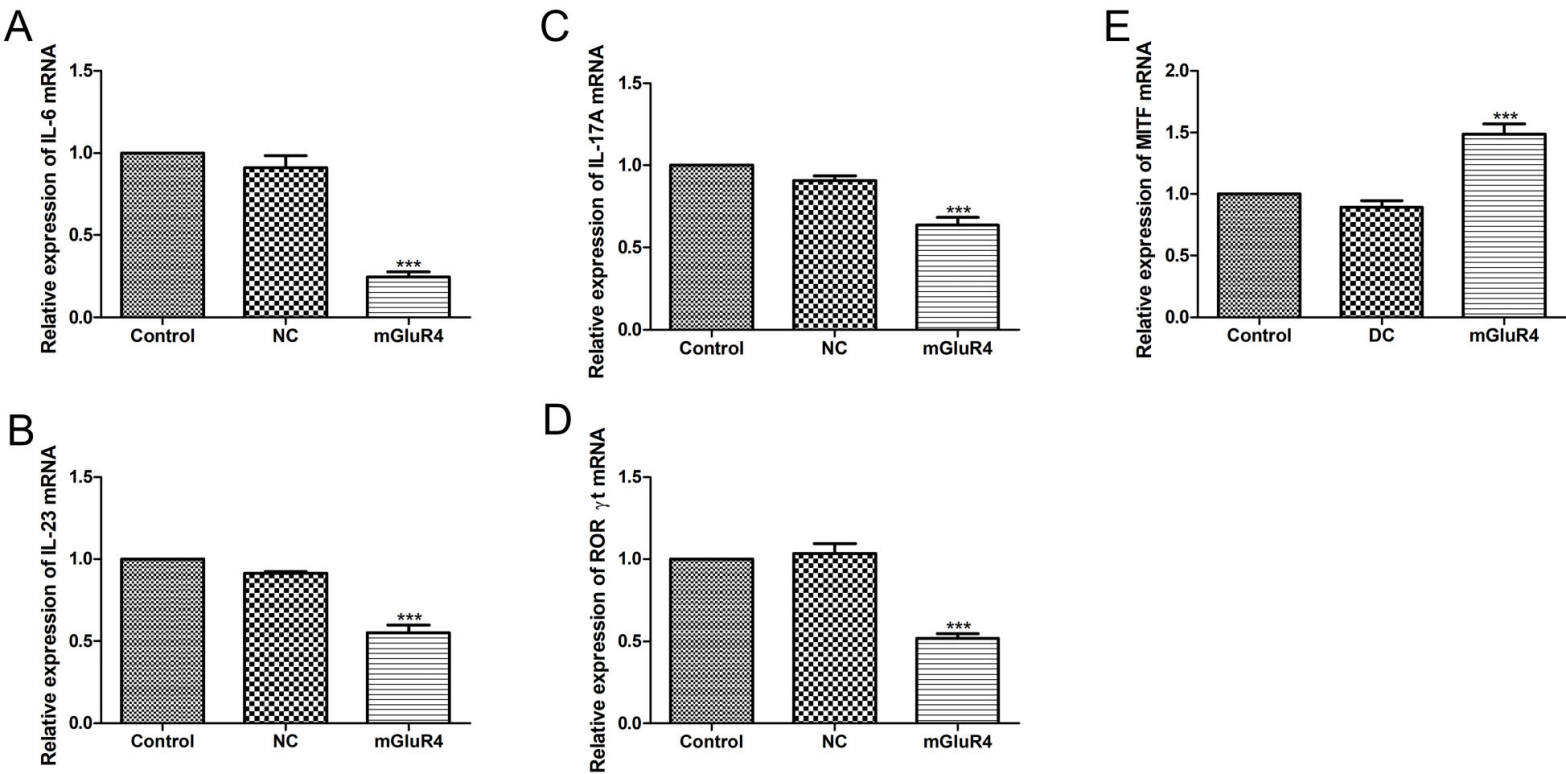
Effect of mGluR4 over-expression on Th17 differentiation and melanogenesis of B16 cells. Dendritic cells (DC) were transfected with empty vector (negative control, NC), plasmid expressing mGluR4 (mGluR4), or untreated (Control). Twenty-four hours later, the relative mRNA expressions of interleukin (IL)-6 (**A**) and IL-23 (**B**) were evaluated by qRT-PCR. CD4^+^ T cells were co-cultured with the above DC for 24 h at the ratio of 1:1. The relative mRNA expressions of IL-17A (**C**) and RORγt (**D**) in co-culture environment were determined by qRT-PCR. B16 cells were treated with co-culture medium of CD4^+^ T cells and empty vector transfected DC (DC), pcDNA3.1 mGluR4 transfected DC (mGluR4), or only RIPM-1640 medium (Control) for 24 h (**E**). mRNA expression of MITF of B16 cells was measured by qRT-PCR. Data are reported as means±SD. ***P<0.001 *vs* Control (ANOVA).

**Figure 8:**
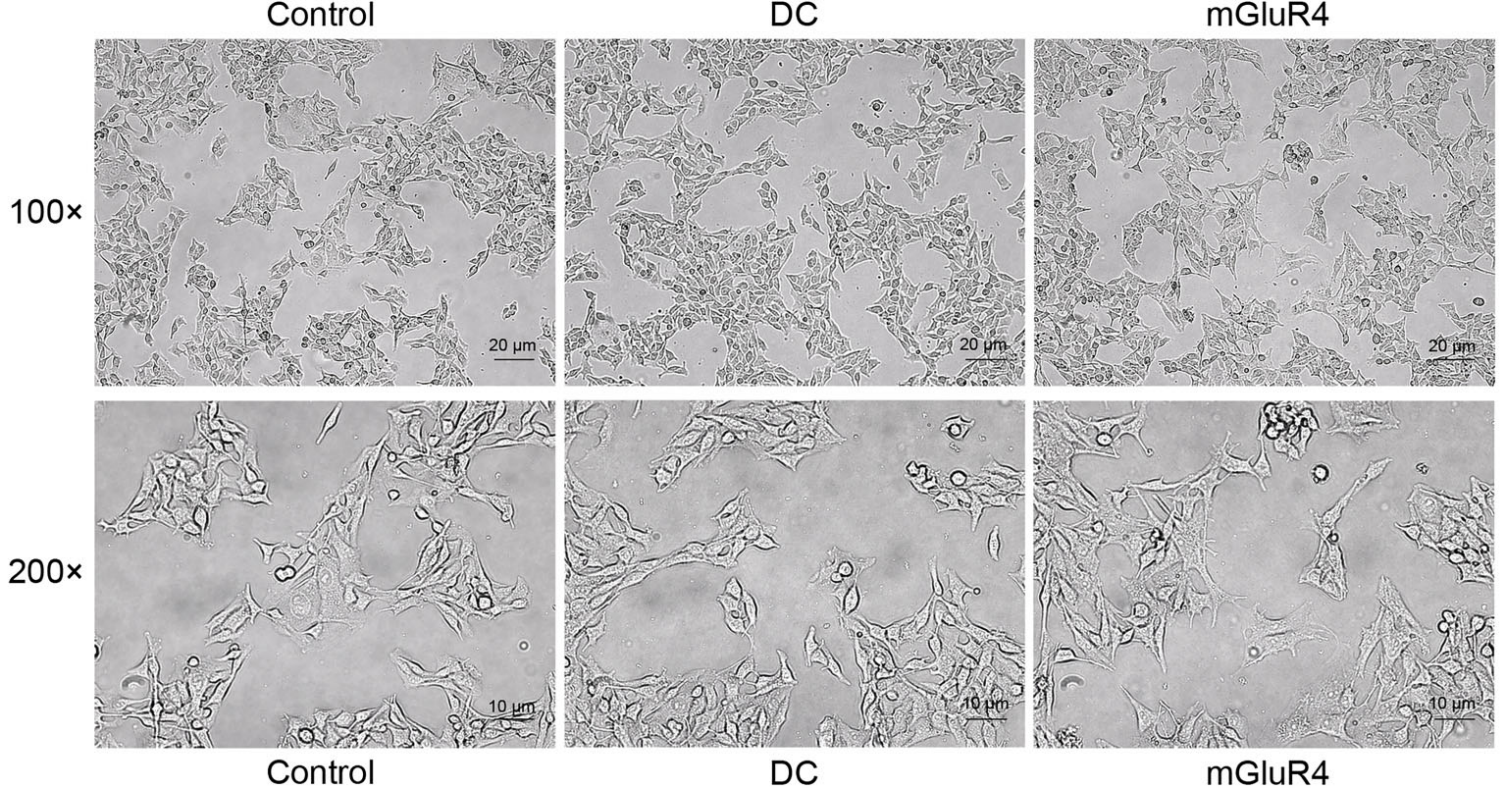
Effect of decreased production of Th17-related cytokines on morphology of B16 cells. B16 cells were exposed to the co-culture medium of CD4^+^ T cells and empty vector transfected dendritic cells (DC), pcDNA3.1 mGluR4 transfected DC (mGluR4), or only RIPM-1640 medium (Control). Twenty-four hours later, melanocyte morphology was observed under the microscope with 100 and 200× magnification (scale bars: upper panels 20 µm; lower panels 10 µm). Representative images are shown.

### mGluR4 knockdown in BMDC induced Th17 cell differentiation

The surface marker expression of costimulatory molecules CD80 and CD86 in DC is critical in stimulating T cell differentiation. We observed no significant differences in the levels of CD80 and CD86 among mature BMDC stimulated with LPS (Control), negative control siRNA transduced BMDC (NC), and mGluR4 siRNA transduced BMDC (siRNA) (data not shown). Then, we investigated whether mGluR4 knockdown in BMDC influenced Th17 differentiation. The expressions of IL-6 (Figure 2A) and IL-23 (Figure 2B) were increased in mature BMDC stimulated with LPS (LPS) compared to that in the immature BMDC (Control) (P<0.001). However, IL-6 (Figure 2A) and IL-23 (Figure 2B) levels were higher in the mGluR4 siRNA transfection group (siRNA) than in the LPS group (P<0.001). Then, we co-cultured the differently treated BMDC with CD4^+^ T cells at the ratio of 1:1. The relative mRNA expressions of IL-17A (Figure 2C) and RORγt (Figure 2D) in the co-culture environment were significantly increased when mGluR4 was knocked down in BMDC (P<0.001). Silencing of mGluR4 in BMDC increased the expression of Th17-related cytokines, inducing Th17 differentiation.

### Increased production of Th17-related cytokines induced by mGluR4 knockdown in BMDC caused dysregulation of melanocyte function

To investigate the direct effects of Th17 cell-related cytokines on melanocytes, we treated B16 cells with co-culture medium of CD4^+^ T cells and the NC siRNA transduced BMDC (DC), mGluR4 siRNA transduced BMDC (siRNA), or only RIPM-1640 medium (Control) (Figure 3). The mRNA expressions of MITF (Figure 3A), TYR (Figure 3B), and TRP-1 (Figure 3C) were significantly decreased in the siRNA group compared to that in the Control group (P<0.001). In addition, the melanin production in the siRNA group was markedly reduced (P<0.001, Figure 3D). After exposure to the co-culture medium of the transduced DC and CD4^+^ T cells, melanocyte morphology was observed under the microscope. The B16 cells were obviously aggregated and varied in shape in the siRNA group, whereas cells in the DC group and the Control group grew with a spindle-shaped morphology (Figure 4).

### cDNA transfection increased the expression level of mGluR4 in BMDC

To further study the effect of mGluR4 on the function of BMDC, we transfected them with empty vector (NC), plasmid expressing mGluR4 (mGluR4), or left them untreated (Control). The mRNA expression and protein level of mGluR4 were increased significantly in the mGluR4 group compared to the Control group by qRT-PCR and western blotting analysis (Figure 5, P<0.001, P<0.01). These results showed that the mGluR4 levels in BMDC were over-expressed by cDNA transfection as expected.

### Over-expression of mGluR4 inhibited Th17 cell differentiation and the reduced production of Th17-related cytokines facilitated the melanogenesis of B16 cells

As shown in Figure 6, the expressions of CD80 and CD86 were decreased in the mGluR4 group compared to the Control group (P<0.001, P<0.05). Subsequently, we investigated whether mGluR4 overexpressed in BMDC affected Th17 differentiation. Our results showed that the expressions of IL-6 (Figure 7A) and IL-23 (Figure 7B) were reduced in the mGluR4 group compared to that in the Control group (P<0.001). Next, we co-cultured the differently treated BMDC with CD4^+^ T cells at the ratio of 1:1. The relative mRNA expressions of IL-17A (Figure 7C) and RORγt (Figure 7D) in the co-culture environment were decreased when mGluR4 was overexpressed in BMDC (P<0.001). mGluR4 over-expression inhibited Th17 differentiation by reducing the expression of Th17-related cytokines. Then, we treated B16 cells with co-culture medium of CD4^+^ T cells and empty vector transfected BMDC (DC), pcDNA3.1 mGluR4 transfected BMDC (mGluR4), or only RIPM-1640 medium (Control) in order to explore the effect of Th17 cell-related cytokines on the function of melanocytes. The mRNA expression of MITF (Figure 7E) was significantly increased in the mGluR4 group compared to the Control group (P<0.001). Additionally, we found that the B16 cells were spindle-shaped in the mGluR4 group similar to that in the DC group and the Control group (Figure 8).

## Discussion

Glutamate functions as a principal excitatory neurotransmitter in the mammalian central nervous system (CNS). Ionotropic glutamate receptors (iGluRs) and mGluRs are the two major classes of glutamate receptors, each of which is further subdivided to specific subtypes or groups containing multiple subunits. mGluRs are localized in the proximity of the synaptic cleft where they regulate neurotransmission, the release of neurotransmitters, and neuronal excitability (17). Recent studies have shown that mGluRs are also widely distributed outside the CNS such as in immune cells (18). mGluR4 is one of the members of Group III mGluRs. In our previous study, we found for the first time that mGluR4 was constitutively expressed in mouse BMDC (16).

DC are the most significant cell type to bridge innate and adaptive immunity. The costimulatory molecules are upregulated and cytokines that drive T cell differentiation are produced when DC are activated (19). Inhibition of DC activation can induce the secretion of regulatory cytokines, shifting the Th cell differentiation away from Th17 phenotype (20). Park et al. (21) reported that APC deficient in CD80 and CD86 failed to bias T cell commitment to the Th17 phenotype. Huang et al. (22) also showed that the blockage of CD86 function suppressed DC-dependent Th17 cell differentiation *in vitro*. In the present study, we found that mGluR4 knockdown in DC did not influence the CD80 and CD86 upregulation after LPS stimulation (data not shown), while the expressions of CD80 and CD86 were decreased in mGluR4 over-expression DC. The result indicated the effect of mGluR4 on the activation of DC.

Naive CD4^+^ T helper cells differentiate into distinct lineages that produce signature cytokines, upon encountering the foreign antigens and costimulatory molecules presented by DC. These differentiation programs, characterized by the expression of lineage-specific transcription factors, are primarily shaped by cytokines produced by DC (10). Th17 cells producing IL-17 have critical functions in the host defense response against extracellular pathogens, as well as the pathogenesis of numerous autoimmune and allergic disorders (23). TGF-β in the presence of IL-6 or IL-21 initiates Th17 cell differentiation (10). Since the IL-23 receptor is absent on naive CD4^+^ T cells and rapidly upregulated after the initiation phase, it is crucial for the expansion and maintenance of Th17 cells (24). Transcription factors RORγt and RORα are highly induced in Th17 cells and play pivotal roles in specifying lineage differentiation (25).

Our previous study has shown that silencing of mGluR4 in BMDC significantly increases IL-17A and RORγt expression in co-culture environment (16). In the present study, we further found the relatively lower expressions of IL-6 and IL-23 in immature BMDC, along with the relatively lower expressions of IL-17 and RORγt in the co-culture system containing immature BMDC compared to that in the mature BMDC stimulated by LPS. Moreover, we observed the increased levels of those cytokines in BMDC with mGluR4 knockdown. Conversely, the expressions of the above Th17-related cytokines were notably reduced in the mGluR4 over-expression group. Fallarino et al. (15) also reported that cDC and pDC from mGluR4-knockout mice secreted more IL-6 and IL-23 than their wild-type counterparts in the presence of LPS or CpG-ODN, respectively. Repeated *in vivo* administration of PHCCC (a selective enhancer of mGluR4) led to an attenuated Th17-mediated immunity. Taken together, these findings indicated that mGluR4 appeared to be a potential regulator of Th17 differentiation. It has been reported that mGluR4 activation reduces intracellular cAMP production in a Gi protein-dependent way (26). Pathways increasing cAMP accumulation in DC facilitate the production of Th17-related cytokines (such as IL-6 and IL-23) (27). Therefore, we conclude that the effect of mGluR4 on Th17 cell differentiation might be involved in the modulation of cAMP in DC. We will further explore this mechanism in the future.

Vitiligo is a common autoimmune hypo-pigmentary skin disorder, caused by selective destruction of melanocytes (28). Recent study demonstrated enhanced Th17 response in peripheral blood of patients with vitiligo (5). IL-17 is dramatically correlated with the progression and severity of vitiligo (6). Abdallah et al. (29) reported that IL-6 was the most sensitive serum marker to distinguish active from stable vitiligo. Vaccaro et al. (30) reported that IL-23 serum levels were notably higher in patients with vitiligo than controls, and positively correlated with disease duration, disease activity, and the extent of vitiligo. These above studies suggest that Th17 cells participate in the pathogenesis of vitiligo. However, the specific role of Th17 cells in melanocytes has yet to be elaborated.

MITF is the major transcription factor that regulates melanocyte fate and melanogenic activity (31). The expression of MITF is significantly decreased in lesion and perilesion skin sections of vitiligo (31). TYR and TRP-1 are critical for the synthesis of melanin, the loss of which contributes to the development of vitiligo (32). Recent study has shown that cytokine imbalance in systemic and skin microenvironments is closely associated with melanocyte biology and vitiligo pathogenesis (28). IL-6 secreted by adjacent keratinocytes in epidermal melanin unit inhibits the growth and proliferation of melanocytes in a paracrine manner (33). Kamaraju et al. (34) have reported that IL-6 inhibits melanogenesis in melanoma cells through downregulating the expression of MITF-M and TYR. Choi et al. (35) have found that IL-6 decreases melanogenesis and the transcription of MITF in normal human melanocytes. Kamaraju et al. (34) have found that the MITF promoter activity is silenced via IL-6/IL-6R signaling mediated by the downregulation of Pax3. Kotobuki et al. (4) have proven that IL-17 induces the production of IL-1β, IL-6, and TNF-α in skin-resident cells such as keratinocytes and fibroblasts, and adversely affects the function of melanocytes.

We observed that the MITF, TYR, and TRP-1 levels and the melanin content of B16 cells were significantly decreased, and most cells were destroyed when B16 cells were treated with the co-culture medium of the mGluR4 knockdown DC and CD4^+^ T cells. However, the expression of MITF was markedly increased and cells were spindle-shaped in the mGluR4 over-expression group. Our results showed that mGluR4 knockdown or over-expression in BMDC influenced the production of Th17-related cytokines, leading to the alteration of melanin content and morphology as well as MITF, TYR, and TRP-1 levels in B16 cells. To distinguish which type of cytokine expressed by Th17 or BMDC is involved in the melanogenesis of B16 cells, cytokine antibody blocking test should be done to block the function of determined cytokines in co-culture medium. We will investigate this mechanism in the future.

Our results were similar to those of Kotobuki et al. (4) that demonstrated the reduced expression of MITF and its downstream genes and the decreased melanin production in HeMnMP (a moderately pigmented human melanocyte cell line) treated with Th17-related cytokines: IL-17A, IL-1β, IL-6, and TNF-α. Therefore, we concluded that the Th17 cell-related cytokines environment modulated by downregulation or upregulation of mGluR4 in BMDC affected the expression of MITF and its downstream genes, along with the morphology and melanin production in B16 cells, leading to the local pigment abnormity. Recently Zhou et al. (36) found that IL-17 promoted the autophagy-mediated apoptotic process, which contributed to the pathology of vitiligo. In a future study, it is suggested to probe into whether the conditioned media for CD4 co-culture experiment would impact cell cytotoxicity, cell cycle, etc., of B16 cells, to determine how the Th17 cytokines would destroy B16 cells. We will carry out the experiment in subsequent research.

In conclusion, we found that increased production of Th17-related cytokines induced by knockdown mGluR4 signaling pathway gave rise to a decreased expression of MITF and its downstream genes, as well as a reduced melanin content and destruction of B16 cells. However, the over-expression of mGluR4 did the opposite. Our research provided new evidence of the role of mGluR4 in the Th17-mediated immune-pathogenesis of vitiligo, which will facilitate the development of promising targeted therapies to intervene with refractory vitiligo.
